# Effect of delayed misoprostol dosing interval for induction of labor: a retrospective study

**DOI:** 10.1186/s12884-019-2454-9

**Published:** 2019-08-27

**Authors:** Elizabeth H. Harman Crowell, Alexander M. Crowell, Regan N. Theiler

**Affiliations:** 1 Dartmouth Hitchcock Medical Center, Lebanon, USA; 2 Geisel School of Medicine at Dartmouth, Hanover, USA; 3 Department of Obstetrics and Gynecology, Mayo Clinic, 200 First Street SW, Rochester, MN 55905 USA

**Keywords:** Cesarean section, Induction, Misoprostol, Multiparty, Nulliparity, Vaginal delivery

## Abstract

**Background:**

Induction of labor occurs in greater than 22% of all pregnancies in the United States. Previous studies have shown that misoprostol is more effective for induction than oxytocin or dinoprostone alone. The World Health Organization recommends vaginal misoprostol 25mcg every 6 hours and the American Congress of Obstetricians and Gynecologists recommends 25mcg vaginal misoprostol every three to 6 hours. Although route of administration and dosage of misoprostol has been extensively studied, little is known about the optimal dosing interval of vaginal misoprostol.

**Methods:**

The primary objective of this study is to determine the effect of delayed vaginal misoprostol dosing, defined as any interval longer than 4.5 h, on time to vaginal delivery. Our hypothesis is that the routine dosing interval of 4 hours shortens times to vaginal delivery compared to delayed dosing, even when adjusted for the time of delay. Secondary objectives include the effect of delayed vaginal misoprostol dosing on cesarean section rate, operative vaginal delivery rate, maternal outcomes, and neonatal outcomes.

We conducted a retrospective chart review of 323 inductions of labor at one academic institution. The primary outcome was the proportion of patients who achieved a vaginal delivery within 24 h. The group who received all doses of misoprostol within a 4.5 h dosing window (Routine Dosing Interval Group) was compared with the group who had any dosing deviation (Delayed Dosing Interval Group).

**Results:**

Of 133 included patients, 64 subjects received routine interval dosing and 69 subjects received delayed interval dosing. The vaginal delivery rates within 24 h were 56% (36/64) and 20% (14/69), respectively (*P* < 10^− 4^). Spontaneous vaginal delivery rates were 86% (55/64) vs. 75% (52/69), respectively (*P* = .13). Kaplan Meier curves demonstrated statistically significant difference in time to vaginal delivery between groups, with a Cox Proportional Hazard ratio for routine dosing interval of 1.73 (*P* < 10^− 5^) unadjusted and 1.34 (*P* = .01) when adjusted for dosing delay.

**Conclusions:**

This retrospective study demonstrates a significant increase in delay-adjusted time to vaginal delivery when doses of vaginal misoprostol are delayed past 4.5 h.

**Electronic supplementary material:**

The online version of this article (10.1186/s12884-019-2454-9) contains supplementary material, which is available to authorized users.

## Background

Induction of labor occurs in greater than 22% of all pregnancies in the United States [1]. This number is growing in association with an increased incidence of obesity, advanced maternal age, and maternal comorbidities [1]. Historically, oxytocin, nipple stimulation, and amniotomy have been used for induction of labor, but more recently cervical ripening has been found to shorten time to vaginal delivery. Previous studies have shown that misoprostol is more effective than oxytocin or dinoprostone alone [2, 3].

Misoprostol can be given either vaginally or orally for cervical ripening [4]. Khan et al found that oral administration of misoprostol has a shorter time to onset and shorter half-life than vaginal misoprostol, but vaginal misoprostol has been found to cause fewer side effects such as nausea, vomiting, and cramping [5, 6].

Other research that has focused on the optimal dose of misoprostol, showing that delivery is expedited with a dose of 50 mcg vaginal misoprostol every 4 hours [2]. However, a Cochrane review concluded that the risk of adverse fetal outcomes such as tachysystole, neonatal intensive care unit admissions, and meconium stained amniotic fluid outweighs the benefits of higher doses [7]. Although route of administration and dosage have been well-studied, little is known about the optimal misoprostol dosing interval.

The World Health Organization recommends vaginal misoprostol 25mcg every six hours and the American Congress of Obstetricians and Gynecologists recommends 25 mcg vaginal misoprostol every three to six hours [1, 8]. However, little available literature supports these administration intervals. Our objective was to determine the effect of vaginal misoprostol dosing interval on time to vaginal delivery and to examine the clinical correlates of delayed dosing intervals. Our hypothesis was that a 4 hour dosing interval of vaginal misoprostol shortens time to vaginal delivery compared to longer interval dosing.

## Methods

We conducted a retrospective chart review of all inductions of labor at Dartmouth-Hitchcock Medical Center from April 2013 to December 2015. The study was approved by the Dartmouth College Center for Protection of Human Subjects (Institutional Review Board). Subjects included were females undergoing induction of labor at our center who were greater than 30 weeks gestational age with intact membranes. They must have received two or greater doses of vaginal misoprostol, and must not have received any oral misoprostol or vaginal dinoprostone.

The primary outcome was the proportion of patients who achieved a vaginal delivery in less than 24 h from initiation of induction with vaginal misoprostol. A power calculation was performed based on a previously reported 66% vaginal delivery rate within 24 h after induction of labor with 25mcg vaginal misoprostol as determined by Elati et al [4]. A reduction in vaginal deliveries within 24 h from 66 to 41% (25 percentage points) was deemed to be clinically significant. A corresponding power calculation (OpenEpi, Version 3) with 80% power and a confidence interval of 95% required a total sample size of 134 women. Data was collected retrospectively in reverse chronological order from December of 2015 until 134 women met inclusion criteria. In the final analysis 133 women were included because one patient had received misoprostol 3 hours after her first dose, which was outside of protocol.

The delivery summary data was imported from the electronic health record (EHR), and elements confirmed by manual review. Medication data was extracted from the medication administration record and misoprostol dosing interval was calculated manually. Progress notes and delivery summaries were reviewed to determine reasons for medication delay, and to review labor course. Data was then placed into a REDCap database.

At our institution vaginal misoprostol is ordered for administration every 4 hours. We allowed an additional 30 min buffer for the routine dosing group. Women who received all doses of misoprostol within the 4.5 h interval, the routine dosing group, were compared with women whose dose administration was delayed beyond 4.5 h. Continuous variables were compared with two-sided Student’s T-Tests and ordinal variables were compared with Chi Squared Tests or Fischer’s Exact Tests based on group size. Time to vaginal delivery was compared using Kaplan Meier curves constructed using Python’s Lifelines Package v0.13 [9]. Cesarean section deliveries were censored out of the Kaplan Meier curve at the time of the event. Confidence intervals were determined by the Kaplan Meier estimator, and hazard rates as well as significance levels for each covariate were determined with a Cox Proportional Hazard Model.

## Results

**Fig. 1:**
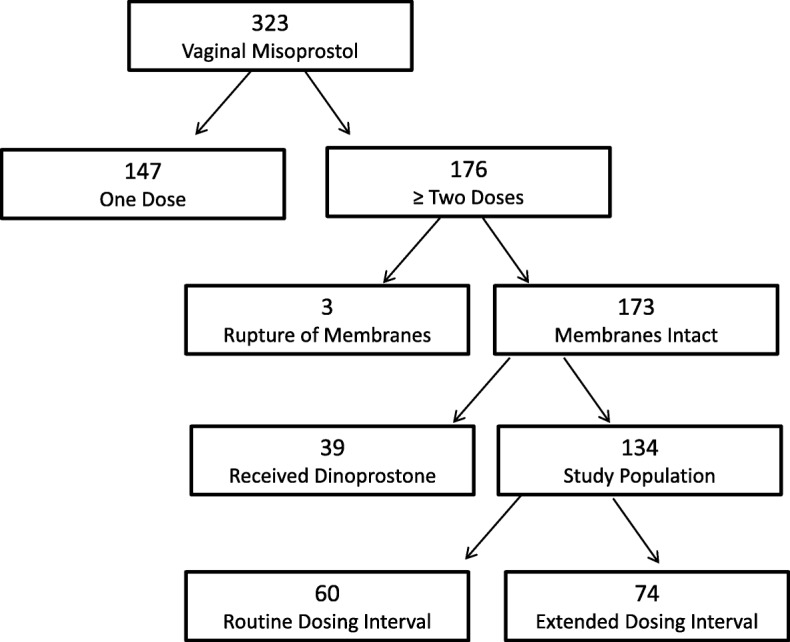
Selection of our study population based on exclusion and inclusion criteria

Of 323 charts reviewed, 134 patients met original inclusion criteria. Sixty-four subjects received routine dosing and 70 subjects received delayed interval dosing (Fig. 1). An outlier was excluded as one of the patients was incorrectly categorized in the delayed interval dosing group when she received her second dose only 3 hours after her first dose. The total group analyzed was 133 patients. The groups were similar, with a significant difference only in parity (Table 1). Fifty percent of the patients in the routine interval dosing group had had a prior vaginal delivery, which was significantly higher than 32% in the delayed interval dosing group (*P* = .036). Induction indications and starting Bishop scores were not significantly different between groups.

**Table 1:** Characteristics of women undergoing induction of labor

Demographics	Routine Interval Dosing, *n* = 64	Delayed Interval Dosing, *n* = 69
Maternal Age, Yr	30.5 (+/− 7.06)	29.9 (+/− 6.3)
Maternal BMI, kg/m^2^	33.7 (+/− 8.02)	35.3 (+/− 7.35)
Gestational Age, weeks	39 + .73 days (+/−14.29 D)	38 + 5.9 days (+/− 15.5 D)
Median Starting Bishop Score	3	2
White (Non Hispanic)	98.44%	95.7%
Non-smoker	87.50%	91.3%
Drug Use	7.8%	4.3%
Prior Vaginal Delivery	50%*	31.9%*
Oxytocin Administration	71%	71%
Induction Indications		
Hypertensive Disorder	32.81%	39.1%
Late Term Gestation	31.25%	24.6%
AMA	6.25%	4.3%
Diabetes	7.81%	8.7%
Cholestasis	1.56%	4.3%
IUGR	7.81%	7.2%
Oligohydramnios	1.56%	4.3%
Other	10.94%	5.7%
Unstable Lie	0	1.4%

Demographics of the study population. Patients in the delayed interval dosing group had at least one dosing interval longer than 4.5 h. Categorical variables are reported as percentage and continuous variables are reported as mean (+/− standard deviation) unless otherwise noted. *P < 0.05

Vaginal delivery rates were 56% (*n* = 36/64) and 20% (*n* = 14/69) within 24 h in the routine dosing vs. delayed interval dosing groups, respectively (*P* < 10^− 4^). When stratifying by patients who received only 2–3 doses of misoprostol, the vaginal delivery rate was 61% (*n* = 36/59) and 29% (*n* = 12/41) within 24 h (*P* = .004). Cumulative spontaneous vaginal delivery rates were not significantly different between the routine vs. delayed interval dosing groups, 86% (*n* = 55/64) vs. 75% (*n* = 52/69) with a *P* = 0.13 (Table 2), although the study was not powered to detect a difference in secondary outcomes. Oxytocin administration was similar, with 71% of patients in each group receiving Oxytocin infusions. No significant differences in outcomes for the neonate or the mother were observed when comparing the two groups (Table 2).

**Table 2:** Secondary maternal and neonatal outcomes

Outcome	Routine Dosing *n* = 64	Delayed Dosing *n* = 69	*P* Value
Vaginal Delivery	55 (86%)	52 (75%)	*P* = .13
Operative Vaginal Delivery	0	6 (8.6%)	N/A
Cesarean Delivery	9 (14%)	12 (18.8%)	*P* = .81
Estimated Blood Loss	455 mL (+/− 348.14)	489 mL (+/− 357.6)	*P* = .58
Post-Partum Hemorrhage	10 (15.63%)	15 (21.7%)	*P* = .39
Meconium	6 (9.38%)	7 (10.1%)	*P* = 1.0
Chorioamnionitis	1 (1.56%)	0	N/A
Shoulder Dystocia	2 (3.13%)	0	N/A
1 min APGAR	7.6 (+/− 1.66)	7.5 (+/−1.95)	*P* = .75
5 min APGAR	8.7 (+/− .87)	8.6 (+/− 1.05)	*P* = .55
NICU stay	4 (6.25%)	10 (14.5%)	*P* = .16

Maternal and neonatal outcomes at delivery by dosing interval. Patients in the delayed interval dosing group had at least one dosing interval longer than 4.5 h. Categorical variables are reported as n, (%), and were compared using chi squared or Fischer’s exact tests. Continuous variables are reported as mean (+/− standard deviation), and were compared using Student’s T-test

The delayed interval dosing group required more doses of misoprostol, a mean of 3.41 vs 2.38, respectively (*P* < 10^− 4^). Reasons cited for delay of administration included inadequate nurse to patient ratio, frequent contractions, patient choice, tachysystole, and non-reassuring fetal heart tracings (Additional file 1: Table S1). Only one patient had documentation of tachysystole with non-reassuring tracing, and that patient was in the delayed dosing group. Additional dosing interval details are shown in Additional file 2: Figure S1.

**Fig. 2:**
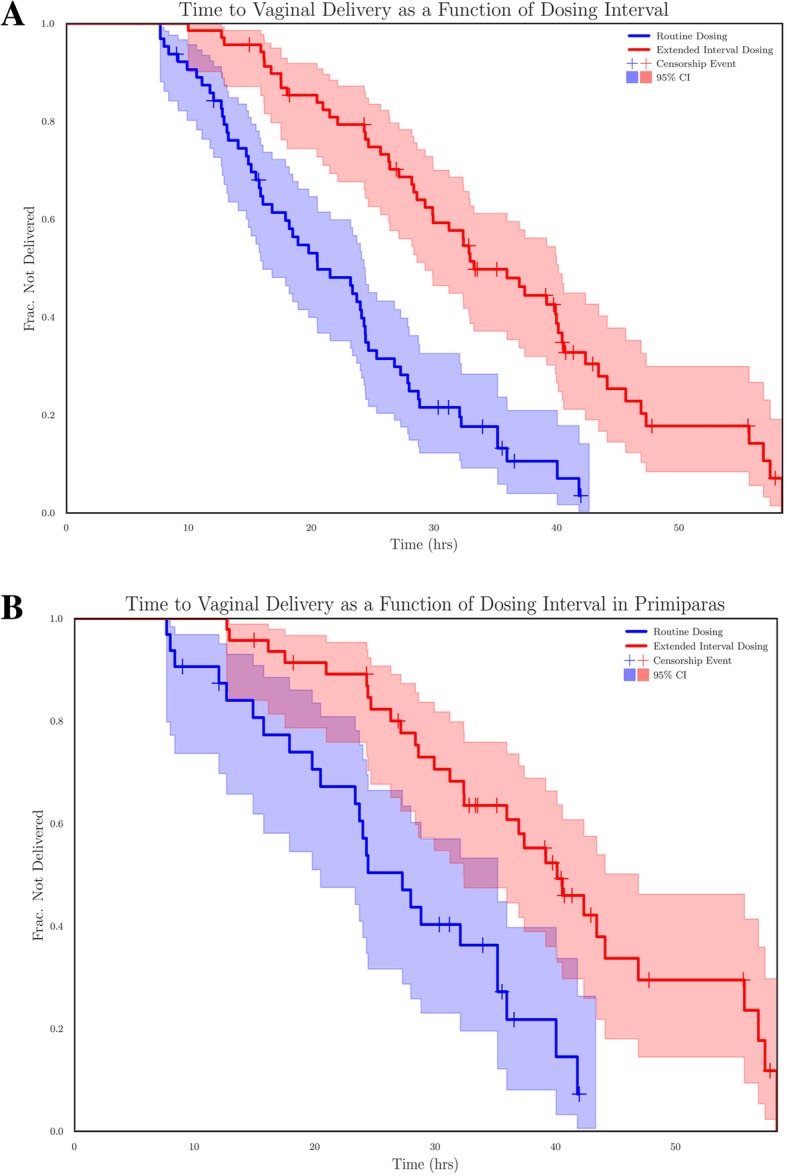
Kaplan Meier Curves showing proportion of patients undelivered vs. time, by misoprostol dosing interval. Shaded areas represent the 95% confidence interval as calculated by the Kaplan Meier Curve Estimator. Crosses represent patients censored for cesarean delivery or vacuum assisted vaginal delivery. **a** demonstrates a significant difference in the hazard ratio even when delay of misoprostol administration was accounted for. **b** is stratified by parity, and Cox Proportional Hazard Ratio for each of the above covariates is significant, with *P* values less than 0.05 (see Table 3)

Kaplan Meier curves demonstrated a statistically significant difference in time to vaginal delivery between groups, with adjusted and unadjusted Cox Proportional Hazard ratios of 1.73 (*P* < 10^− 5^) and 1.34 (*P* = .01) for the time to vaginal delivery in the routine dosing group (Table 3). The adjusted curve subtracts the time attributable to the dosing delay from the time to vaginal delivery (Fig. 2a). We further controlled for parity by demonstrating that the Cox proportional hazard ratio for delayed interval dosing was greater than 1 in both the adjusted and unadjusted Kaplan Meier Curves (Fig. 2b, Table 3).

**Table 3:** Cox proportional hazard ratios corresponding to Kaplan-Meier curves in Fig. 2. The adjusted curve corrects for the time attributable to the dosing delay. Both adjusted and unadjusted values are shown

Covariates	Cox Proportional Hazard Ratio	*P* Value
Prior Vaginal Delivery (Unadjusted)	1.59	.0002
Prior Vaginal Delivery (Adjusted)	1.65	.00008
Dosing Interval (Unadjusted)	1.73	.000004
Dosing Interval (Adjusted)	1.34	.01

### Comment

This retrospective study demonstrates an increase in time to vaginal delivery when doses of vaginal misoprostol are delayed past 4.5 h. This was demonstrated not only by the primary outcome of a 56 and 20% spontaneous vaginal delivery rate within 24 h (*P* < 10^− 4^) in the routine dosing vs. delayed interval dosing group, but also in a sub-analysis of those patients who had only received two or three doses of misoprostol. Stratifying by number of doses misoprostol received allowed us to control for the delay in time to vaginal delivery attributable to the non-intervention period between each dose of misoprostol, and the difference between the two groups remained statistically significant. Although dosing interval has not been previously studied in inductions of term labor, the Society of Family Planning also concluded that dosing interval of vaginal misoprostol in second trimester abortions is at least as important as dose for timely delivery [10].

The delay-adjusted Kaplan Meier curve suggests that the delay in time to vaginal delivery is greater than that which could be attributed to delay in medication administration alone. The starting Bishop Scores of this patient population are much lower than the previous thresholds designated as a favorable cervix, > 8 or more conservatively > 5, and were not significantly different between study groups [11, 12]. The delayed interval group contained significantly more multiparous patients, which we controlled for by stratifying by parity, Fig. 2b. This suggests that 4 hour interval dosing of vaginal misoprostol initiates the cascade of labor more effectively than delayed interval dosing. This is consistent with the study by Khan et al. describing the peak concentration and half-life of vaginal misoprostol [5]. Patients who received 4 hour interval dosing of vaginal misoprostol not only had a higher likelihood of a vaginal delivery within 24 h, but also a shorter time to delivery.

This study is unique as we examine the dosing interval of misoprostol, rather than the route of administration or dose. This study did, however, have some limitations. By conducting a retrospective cohort study we were unable to control for starting bishop score, parity, and other factors known to influence time to vaginal delivery. Although the study included a variety of patients, the homogeneity of our academic institution with 98.5% Caucasian patients may make this study less applicable to more diverse populations.

Vaginal delivery rates within 24 h were lower in our study, compared to the study by Elati [4]. This may be secondary to the 25mcg dose of vaginal misoprostol that was studied and the exclusion of patients who only received one dose of vaginal misoprostol in our cohort. It has been shown that induction with 50mcg doses of vaginal misoprostol had the highest rate of delivery within 24 h [2, 7, 13].

We present preliminary evidence that adherence to every 4 hour dosing of vaginal misoprostol leads to an increased rate of vaginal delivery within 24 h, and that delayed doses result in need for more doses of misoprostol and longer time to delivery. Efforts should be taken to adhere to efficacious methods of induction of labor to improve outcomes for both the mother and child. As our study was not powered to detect adverse maternal and fetal outcomes or rates of operative delivery, future randomized controlled trials are needed to define optimal dosing regimens for induction of labor using vaginal misoprostol.

## Additional files

Additional file 1: **Table S1.** Indications for misoprostol dose delay among 69 patients. Frequent contractions were defined as regular uterine contractions that did not meet criteria for tachysystole. Patient choice indicates request of the patient to delay dose, and floor acuity refers to staffing ratios being inadequate to continue induction of labor. Non-reassuring fetal status is defined as persistent category II or category III fetal heart monitoring. Doses, *n* (%) refers to the number and percentage of delayed misoprostol doses. (DOCX 13 kb)

Additional file 2: **Figure S1.** Individual between-dose intervals for routine (blue) and delayed (red) administration groups. (JPG 2256 kb)

## Data Availability

The datasets used are available from the corresponding author on reasonable request.

## Abbreviations

EHR: Electronic Health Record
Mcg: Micrograms
REDCap: Research Electronic Data Capture
