# Supplementary material for: Effect of delayed misoprostol dosing interval for induction of labor: a retrospective study
Source: BMC Pregnancy Childbirth. 2019 Aug 27;19:309. doi: 10.1186/s12884-019-2454-9 (PMC6712846; doi:10.1186/s12884-019-2454-9)
Supplement: Supplementary file 1 — Table S1. Indications for misoprostol dose delay among 69 patients. Frequent contractions were defined as regular uterine contractions that did not meet criteria for tachysystole. Patient choice indicates request of the patient to delay dose, and floor acuity refers to staffing ratios being inadequate to continue induction of labor. Non-reassuring fetal status is defined as persistent category II or category III fetal heart monitoring. Doses, n (%) refers to the number and percentage of delayed misoprostol doses. (DOCX 13 kb) [file 12884_2019_2454_MOESM1_ESM.docx]

**Additional file 1: Table S1**

| **Reason for Delay** | **Doses, n (%)** |
| --- | --- |
| Frequent Contractions | 20 (21.5) |
| Patient Choice | 16 (17.2) |
| Floor Acuity | 9 (9.7) |
| Tachysystole | 2 (2.2) |
| Non-Reassuring Fetal Status | 1 (1.1) |
| Not Documented | 43 (46.2) |
| Total | 93 (100) |

**Table 1.** Indications for misoprostol dose delay among 69 patients. Frequent contractions were defined as regular uterine contractions that did not meet criteria for tachysystole. Patient choice indicates request of the patient to delay dose, and floor acuity refers to staffing ratios being inadequate to continue induction of labor. Non-reassuring fetal status is defined as persistent category II or category III fetal heart monitoring. Doses, n (%) refers to the number and percentage of delayed misoprostol doses.
